# Diagnostic performance of two rapid tests for syphilis screening in people living with HIV in Cali, Colombia

**DOI:** 10.1371/journal.pone.0282492

**Published:** 2023-03-09

**Authors:** Jonny Alejandro García Luna, Nelson Romero-Rosas, Sebastian Alejandro Silva Peña, Oscar Javier Oviedo Sarmiento, Ximena Galindo Orrego, William Lenis Quintero, Luisa Consuelo Perea, Ernesto Martínez Buitrago, Lyda Osorio, Juan Carlos Salazar, Adrian D. Smith, Neal Alexander

**Affiliations:** 1 Centro Internacional de Entrenamiento e Investigaciones Médicas – CIDEIM, Cali, Colombia; 2 Universidad Icesi, Cali, Colombia; 3 Division of Dermatology and Dermatologic Surgery, Department of Internal Medicine, School of Medicine, Universidad del Valle, Cali, Colombia; 4 Corporación de Lucha Contra el SIDA, Cali, Colombia; 5 Clínica Recuperar IPS, Cali, Colombia; 6 Division of Infectious Diseases, Department of Internal Medicine, School of Medicine, Universidad del Valle, Cali, Colombia; 7 Grupo Colombiano de VIH (VIH-COL), Cali, Colombia; 8 School of Public Health, Universidad del Valle, Cali, Colombia; 9 Department of Pediatrics, University of Connecticut School of Medicine, Farmington, Connecticut, United States of America; 10 Division of Pediatric Infectious Diseases, Connecticut Children’s, Hartford, Connecticut, United States of America; 11 Department of Immunology, University of Connecticut School of Medicine, Farmington, Connecticut, United States of America; 12 Nuffield Department of Population Health, University of Oxford, Oxford, United Kingdom; Food and Drug Administration, UNITED STATES

## Abstract

**Introduction:**

There is insufficient evidence supporting the use of rapid diagnostic tests (RDTs) for syphilis in people living with HIV (PLWH). We evaluated the diagnostic performance of two commercially available RDTs (Bioline and Determine) in PLWH in Cali, Colombia.

**Methods:**

A cross-sectional field validation study on consecutive adults with confirmed HIV diagnosis attending three outpatient clinics. Both RDTs were performed on capillary blood (CB), obtained by finger prick, and sera, by venipuncture. A combination of treponemal enzyme linked immunosorbent assay (ELISA) and *Treponema pallidum* haemagglutination assay (TPHA) on serum samples was the reference standard. Rapid plasma reagin (RPR) and clinical criteria were added to define active syphilis. Sensitivity and specificity, predictive values and likelihood ratios (LR) of RDTs were estimated with their corresponding 95% confidence interval (95% CI). Stratified analyses by sample type, patient characteristics, non-treponemal titers, operator and re-training were performed.

**Results:**

244 PLWH were enrolled, of whom 112 (46%) had positive treponemal reference tests and 26/234 (11.1%) had active syphilis. The sensitivities of Bioline on CB and sera were similar (96.4% vs 94.6%, p = 0.6). In contrast, Determine had a lower sensitivity on CB than sera (87.5% vs 99.1%, p<0.001). Sensitivities were lower in PLWH not receiving ART (Bioline 87.1% and Determine 64.5%, p<0.001) and for one of the operators (Bioline 85% and Determine 60%, p<0.001). Specificities of the RDTs were > 95% in most analyses. Predictive values were 90% or higher. For active syphilis, the RDTs showed a similar performance pattern but with decreased specificities.

**Conclusion:**

The studied RDTs have an excellent performance in PLWH to screen for syphilis and potentially for active syphilis, yet Determine performs better on sera than CB. Patient characteristics and potential difficulties operators may face in acquiring enough blood volume from finger pricks should be considered for the implementation and the interpretation of RDTs.

## Introduction

Syphilis is a chronic, multi-stage, sexually transmitted infection caused by the spirochete *Treponema pallidum* subs. *pallidum* [1]. Syphilis remains a major global health problem causing an estimated 6 million new cases every year [2]. In Colombia, the incidence of venereal syphilis is estimated at 300 per 100,000 person years [3] and the number of cases of maternal and congenital syphilis have increased steadily since 2016 underscoring the high burden of this disease in the population [4]. People living with HIV (PLWH) bear a disproportionate burden of this disease [5], Hence, the recommendation that this population is tested at least annually [6]. In Colombia, data from the national registry of HIV showed that 87.3% of PLWH were screened for syphilis and 22,352 of 106,490 (21%) screened tested positive in 2020 [7].

Routine diagnosis of syphilis is based on two types of serologic assays, treponemal tests (TT) that detect antibodies against *T*. *pallidum* proteins and non-treponemal test (NTT) that detect antibodies against lipid antigens. While TT are positive for life, NTT titers are expected to decrease after treatment. However, a high interobserver variability of NTT has been reported [8]. Hence, their interpretation must be correlated to clinical signs and symptoms and when available, the results of previous syphilis tests [1, 6]. Furthermore, serologic assays for syphilis require skilled personnel and laboratory facilities, thus rapid diagnostic tests (RDTs) have been developed and implemented for the diagnosis of syphilis in low resource settings [9]. There are several kinds of rapid tests for syphilis including single treponemal and non-treponemal tests and as part of simultaneous testing with other sexually transmitted infections. Immunochromatographic testing is the most widely implemented platform to detect treponemal antibodies [10, 11]. Previous meta-analysis of field validation studies of these RDTs have found a sensitivity of approximately 85% with significant heterogeneity and a consistent specificity of more than 90% [10, 11].

RDTs have been implemented at the point-of-care as part of programs for prevention of mother-to-child transmission of syphilis, and have increased coverage and reduced the time to treatment [9]. These tests, have also been implemented within sexually transmitted disease clinics and as part of HIV care programs [12–15]. In Colombia, national HIV care guidelines recommend the use of treponemal RDTs for screening of syphilis in PLWH [16], despite a previous systematic review highlighting the insufficient evidence for their use in this population [10]. The few studies reporting the diagnostic performance of syphilis RDTs in people living with and without HIV have found inconsistent results: a field validation study in Mozambique reported that participants with HIV had higher odds of false negative results in RDTs [17], and a laboratory based validation in Australia found a sensitivity of RDTs >90% in people living with and without HIV, although reported a significant lower specificity in this population compared to people without HIV (>95% vs <89%) [18]. Thus, the aim of our study was to assess the diagnostic performance of two widely used RDTs on serum and capillary blood samples from PLWH in Colombia and to explore potential sources of heterogeneity in performance.

## Material and methods

This manuscript follows the Standard for the Reporting of Diagnostic Accuracy Studies (STARD 2015) guidelines (S1 Table) [19].

### Study design, population and setting

We carried out a cross-sectional validation study with prospective enrolment of consecutive participants at three outpatient study sites: two HIV outpatient clinics (clinic A and B) and the referral clinic of the Centro Internacional de Entrenamiento e Investigaciones Medicas (CIDEIM), in the municipality of Cali in the Southwest of Colombia between July 15^th^ and December 7^th^ of 2021. CIDEIM is a translational research center where patients with untreated early syphilis are referred from public primary health center all over the municipality.

Adults with confirmed HIV diagnosis by means of at least two different HIV tests, who had not been screened for syphilis in the three months prior to the enrolment visit, and who agreed to be part of the study were eligible. In clinic A, consecutive patients were initially evaluated by clinical staff and if they had any indication for blood draw (i.e. infectious diseases screening tests, assessment of metabolic risk or assessment of viral and immunologic status), they were referred to study personnel who assessed the eligibility criteria, performed the informed consent process and the study procedures. In clinic B, consecutive patients attending the blood draw room were assessed by study personnel and included if they met eligibility criteria. Finally, consecutive patients with probable early syphilis who were referred to the CIDEIM clinic from primary health centers in the city were also included in the study if they met eligibility criteria.

### Sample size calculation

Previous laboratory studies suggested a sensitivity of approximately 95% [18]. Using the two tailed Z-score formula [20], we defined a sample size of 79 participants positive by the reference standard to ensure that, for a true sensitivity of at least 95%, there would be 80% power to achieve a lower 95% confidence limit of at least 85%, which was considered as the minimally acceptable [10, 11].

### Index and reference tests

RDTs were selected to include locally commercially available tests approved by the Colombian regulatory authority (Instituto Nacional de Vigilancia de Medicamentos y Alimentos [INVIMA]): SD Bioline Syphilis 3.0 (Standard Diagnostics Inc, Kyonggi-do, Korea) and Alere Determine Syphilis TP (Abbott Diagnostics Medical Co, Ltd). Both tests use an immunochromatographic platform with a strip showing a colored test line if treponemal antibodies are detected in the specimen and a colored control line if the test is working properly. RDTs were run according to manufacturer’s instructions, read after 20 minutes and were considered positive if both the test and control lines were colored, even if the test line was faint. They were considered negatives if only the control line was colored and invalid when only the test or neither of the lines were colored.

RDTs were performed at the point-of-care using capillary blood (CB) from a finger prick and at a reference laboratory using serum. A volume of 20μL of CB or 10μL of serum with its corresponding buffer for Bioline and 50μL of CB with its corresponding buffer or serum without buffer for Determine were used. RDTs on CB were read by one of 5 operators at the point-of-care and, on sera at the reference laboratory by three evaluators, two microbiologists and one physician, RDTs on sera were considered positive or negative when at least two evaluators agreed on the assessment of the test and were considered invalid when at least one evaluator considered the test invalid. Invalid tests were repeated once.

Diagnosis of syphilis is challenging due to the lack of a reliable reference standard; however, serology tests remains as the most widely implemented tests for syphilis [21, 22]. Considering that the index tests under study detect treponemal antibodies, we decided to use two TT as reference standard: *Treponema pallidum* haemagglutination test (TPHA) and enzyme linked immunoassay (ELISA) for syphilis. TPHA (Human Gesellschaft für Biochemica und Diagnostica mbH, Wiesbaden, Germany) and *T*. *pallidum* ELISA (Human Gesellschaft für Biochemica und Diagnostica mbH, Wiesbaden, Germany) were performed using 10 μL of serum for each test. As with index tests, selection of the reference standard kits was based on availability and approval by INVIMA. We decided to use composite reference standard due to the limitations of each individual test such as higher false positive rate of immunoassays and lower sensitivity of agglutination assays during latent syphilis [23]. The reference standard was considered positive when both tests were positive, negative when both tests were negative and undetermined when the two results were discordant or one of the tests was invalid.

### Study procedures

Following informed consent, a targeted physical examination was performed by trained study clinicians (operators 2–5, see below) to identify signs of early syphilis (i.e. rash, ulcers, mucous patches or condyloma lata). An electronic case report form (eCRF) was used to collect demographic (birth date, sex assigned at birth, current gender, ethnicity, area of residency) and clinical information (history of previous syphilis, date of HIV diagnosis and current antiretroviral therapy) via face-to-face or telephonic survey according to the participant time availability. Date and results of the closest (up to 134 days before the enrolment date) viral load (copies/mm^3^) and CD4 cell count (cells/mm^3^) were collected from laboratory results in the corresponding electronic medical record. In clinic A, as part of routine care all patients with newly diagnosed HIV were tested using p24 HIV antigen and CD4 rapid tests. Additionally, patients who had been lost to follow-up and were re-engaged in the HIV care program were also tested for CD4 rapid test. Two samples were obtained from each participant: capillary blood (CB) from finger prick and serum from venous blood. CB was used for RDTs performed at point-of-care and serum for both RDTs and reference tests performed at a reference laboratory.

#### Point-of-care syphilis testing

70μL of CB were obtained by puncture of the lateral aspect of the third or fourth finger of the non-dominant hand using sterile disposable stainless steel blood lancets (MEDIpoint, New York, USA) and collected using EDTA Capillary Tubes (Abbot Diagnostics Medical Co, Ltd). To avoid multiple punctures, RDT were performed using CB from the same puncture using EDTA capillary tubes marked at 70 μL and 20 μL by the microbiologist of which 50 μL were used for Determine and 20μL for Bioline.

RDTs at the point-of-care were performed using CB by five certified operators. Operator 1 was a registered nurse who has been previously certified for rapid testing and was the person responsible for routine rapid testing in clinic A. To avoid multiple punctures, Bioline and Determine RDTs for newly diagnosed and re-engaged HIV cases in clinic A were performed by operator 1 but read by one member of the study staff. Operators 2–5 (study staff) were certified by the local public health laboratory in the performance of RDTs prior to the start of the study. They performed study RDTs using CB at either clinic A, B or CIDEIM and read the RDTs performed by operator 1 in clinic A. Operators 2–4 were physicians and operator 5 was a trained auxiliary nurse, experienced in the care of early syphilis cases. Since RDTs at the point-of-care were performed at the same time for only one participant at a time, operators were not blinded to clinical data or to the results of the other rapid test.

#### Serum collection and handling

When possible, 10 mL of whole blood for serum separation were collected the same day of the RDTs. However, in clinic A the blood draw room was available only on Tuesday and Thursday mornings, and participants who were enrolled on other days of the week had to attend an offsite sample collection room, which was located 10 minutes from the clinic by public transportation. Blood samples were collected through venipuncture using either VACUETTE Multiple Use Drawing Needle 21G (Greiner Bio-One, Monroe, North Carolina) or VACUTAINER Eclipse Blood Collection 21G Needle (BD Diagnostics, Franklin Lakes, New Jersey) by a clinical laboratory technician at the clinics or a microbiologist at CIDEIM. Samples were kept at room temperature (~ 20°C) in either two red top (Vacuette, Greiner Bio-One, Monroe, North Carolina) or two golden top (Vacutainer, BD Diagnostics, Franklin Lakes, New Jersey) serum separator tubes for 30–120 minutes before centrifugation in a standard serum centrifuge (1,000–2,000g) for 10 minutes. Upon centrifugation, serum samples were kept at 2–6°C in a cooler box equipped with continuous monitoring of temperature and 6 ice packs (previously frozen at -20°C for at least 72 hours). Serum samples were transported to the reference laboratory at CIDEIM. Upon arrival at CIDEIM, samples were separated into five, 1 mL aliquots and stored at -80°C within 10 hours of collection. Pre-printed labels with participant study ID codes were stuck to informed consent forms, RDTs strips and serum tubes. The same code was entered into the eCRF to link laboratory, demographic and clinical data.

#### Disease staging

Rapid Plasma Reagin (RPR) (Human Gesellschaft für Biochemica und Diagnostica mbH, Wiesbaden, Germany) was performed using 100 μL of serum. All reactive RPR results were followed by serial serum dilutions to obtain the RPR titer. Participants who were a) positive by either TPHA or ELISA, and b) non-reactive by RPR were considered as no active syphilis. Those with positive TPHA or ELISA and reactive RPR results were also considered as having no active syphilis if RPR titers had a fourfold decrease compared to a previous test, or if they had received syphilis treatment within the last 6 months. Those with positive TPHA or ELISA and reactive RPR results were considered as having active syphilis if signs of primary (chancre on genitals, oral cavity, or perianal region) or secondary syphilis (rash, condyloma lata or mucous patches) were present or if they had a fourfold increase in RPR titers compared to previous results. Serofast status was inferred for those subjects without a fourfold decrease in RPR titers six months after treatment [6].

#### Reference tests and RDTs at reference laboratory

We ensured that serum samples were masked before performing the reference test and RDTs at the reference laboratory. From each participant, a 1 mL serum aliquot was thawed and divided into five aliquots of 200 μL that were stored in pre-labeled tubes with new codes and stored at -80°C until final processing. The new codes differed between aliquots from the same individual and to the study ID and could not be linked by the microbiologist to clinical data. These codes also indicated the test that should be performed with the serum aliquot: either Determine, Bioline, RPR, TPHA and ELISA for *T*. *pallidum*. Since samples of multiple participants were processed at the same time, the microbiologist performed the tests blinded to the results of the other tests and clinical data. Also, the evaluators were blinded to each other results.

### Training and quality control

All tests were stored and performed following manufacturer’s instructions. The five operators attended a 1-hour workshop about the study design and specific instructions for the use of the RDTs in the study. The performance of each operator was monitored comparing the results on CB and sera from the same participant. A second training session to reinforce the RDT procedure was scheduled after the enrolment of 70 participants due to underperformance of operator 1. RDTs were repeated when the results were considered invalid. Furthermore, a subset of RDTs stored in the clinics was transported to the reference laboratory and used with well characterized serum samples to assess any potential detrimental effect of storage conditions in the clinical sites.

The reference tests were performed by a certified microbiologist who standardized them using 40 serum samples, 20 from confirmed early syphilis cases and 20 from healthy donors from previous studies. CIDEIM’s reference laboratory is part of the external quality assurance program monitored by the local public health laboratory. Study staff were instructed to report to the study coordinator any adverse event related to the sample collection and to register in the eCRF the number of finger pricks and venous punctures required for sample collection.

### Statistical analysis

Data was exported from the eCRF and analyzed using R [24] and Stata 16.0 [25]. A descriptive analysis was conducted to identify absolute and relative frequencies of categorical variables and median and range of quantitative variables. Sensitivity, specificity, predictive values and likelihood ratios were estimated for each RDT performed using CB and serum compared to the composite reference standard. Undetermined results in the reference standard were excluded from the analysis. The Wilson score method [26] was used to calculate 95% confidence intervals for operating characteristics using the “diagt” command in Stata. Operating characteristics of RDTs performed on sera were compared to those performed on CB using the package “DTComPair” in R. Sensitivity and specificity of RDTs performed on sera and CB were compared using the exact test for paired data [27]. Predictive values and likelihood ratios were compared using the regression approaches proposed by Leisenring *et* al [28] and Gu *et al* [29] respectively.

To assess the effect of using a composite reference standard [30] sensitivity analyses were performed using alternative reference standards: i) only TPHA results, ii) only ELISA results, iii) composite maximizing the sensitivity, that was considered positive when either was positive, negative when both were negative and invalid when both were invalid. We used active syphilis as an alternative reference standard to assess the sensitivity and specificity of RDTs for active disease, this analysis was restricted to PLWH without previous history of syphilis since this population would be the target population for screening of active syphilis with treponemal RDTs. To explore sources of heterogeneity in the sensitivity and specificity of RDTs, subgroup analyses were performed by sex, age, time since HIV diagnosis, current use of ART, CD4 counts, viral load, previous history of syphilis, RPR results, operators, and re-training. Furthermore, to control for the potential confounding effect of operator 1, stratified analysis of RDTs performed in capillary blood were repeated excluding their results. Subgroups were compared using Fisher’s exact test [20] or the Cuzick test when a linear trend was apparent [31]. Concordance analysis between the summary evaluation of the three evaluators of RDTs on sera was done using Cohen’s kappa with its corresponding 95% CI, using the “kapci” command in Stata. Kappa measures observed agreement above chance, as a proportion of the maximum possible agreement above chance [32]. Landis & Koch’s descriptors for values of kappa were used, e.g. values more than 0.8 were considered as “almost perfect” [33].

### Ethics statement

This study was reviewed, approved, and monitored by the institutional ethical review board of the CIDEIM (reference number: 1302). Written informed consent was obtained from all participants and the study was conducted according to national regulations and the principles of the Declaration of Helsinki. Results of both index and reference test on sera were shared with the clinical staff to assess the need of syphilis treatment of participants. However, considering the potential delay between the sample collection date and the processing of masked sera for reference test, a first RPR was performed on serum samples upon the arrival of the sample to the reference laboratory and their results provided to the treating clinician.

## Results

### Study participants

**Fig 1:**
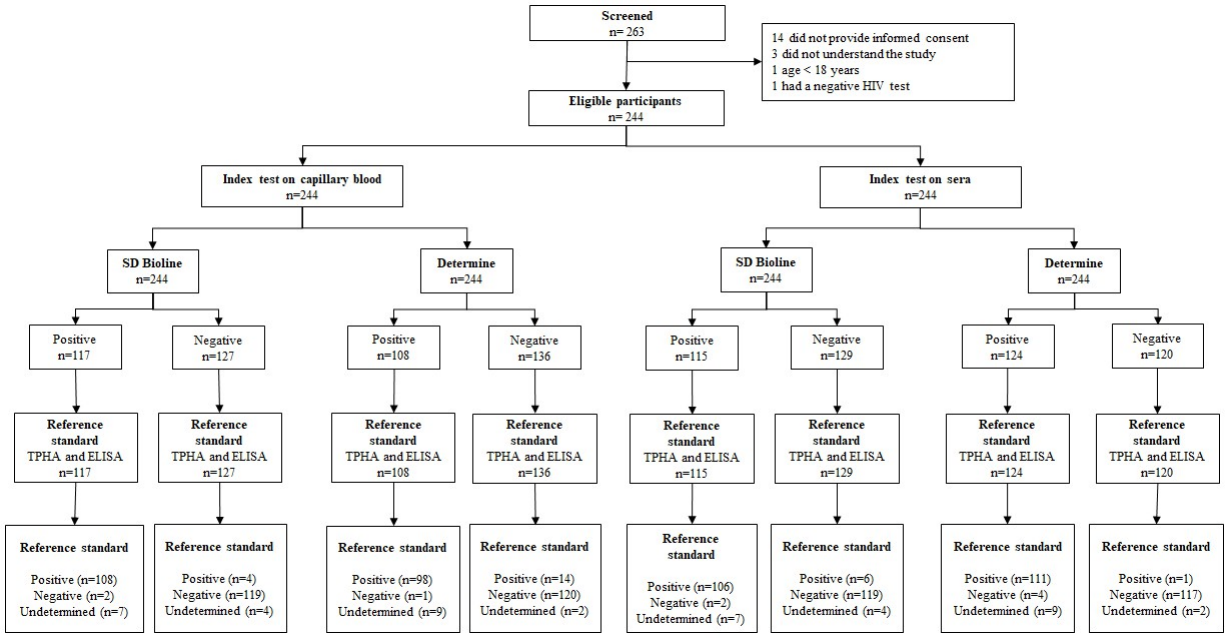
Flow chart of participants.

A total of 263 PLWH were screened and 244 met eligibility criteria. Among the included, 11 had to be excluded from the main analysis due to undetermined results in the reference standard (Fig 1). There were 143 participants enrolled in clinic A, 98 in clinic B and 3 in the CIDEIM referral clinic. Included participants had a median age of 35 years, 72.6% were cis-gender males and 91.0% were residents of urban areas (Table 1).

**Table 1:** Characteristics of study population.

Characteristic	Total (n = 244)^a^
Median age in years (IQR)	35 (27.4–50.2)
18–30 years, n (%)	83 (34.0)
>30 years, n (%)	161 (66.0)
Gender, n = 241	
Cis-gender male, n (%)	177 (73.5)
Cis-gender female, n (%)	54 (22.4)
Trans-gender female, n (%)	9 (3.7)
Non-binary, n (%)	1 (0.4)
Ethnicity, n = 243	
Mixed background, n (%)	133 (54.7)
Afrocolombian, n (%)	62 (25.5)
Indigenous, n (%)	14 (5.8)
White, n (%)	34 (14.0)
Area of residency, n = 243	
Urban, n (%)	222 (91.4)
Rural, n (%)	21 (8.6)
Median time in years since HIV diagnosis (IQR), n = 242	2.5 (0.2–8.6)
≤1 year since diagnosis, n (%)	83 (34.7)
>1 year since diagnosis, n (%)	158 (65.3)
Current antiretroviral therapy	
No, n (%)	77 (31.6)
Yes, n (%)	167 (68.4)
Median CD4 count cells /mm^3^ (IQR), n = 240	405.5 (250.5–612.0)
<200, n (%)	35 (14.6)
200–499, n (%)	123 (51.2)
≥500, n (%)	82 (34.2)
Median viral load copies/mm^3^ (IQR), n = 243	0 (0–16,870)
Undetectable, n (%)	128 (52.7)
≤ 1,000 copies/mm3, n (%)	24 (9.9)
1000–10,000 copies/mm3, n (%)	28 (11.5)
> 10,000 copies/mm3, n (%)	63 (25.9)
Previous history of syphilis n (%), n = 233	
No, n (%)	148 (63.5)
Yes, n (%)	85 (36.5)
RPR titers, n = 244	
Non-reactive, n (%)	172 (70.5)
< 8 dils, n (%)	38 (15.6)
≥ 8 dils, n (%)	34 (13.9)
Syphilis stage, n = 234	
Negative syphilis tests, n (%)	111 (47.4)
No active syphilis, n (%)	95 (40.6)
Primary, n (%)	1 (0.4)
Secondary, n (%)	11 (4.7)
Early latent, n (%)	8 (3.4)
Late latent, n (%)	6 (2.6)
Serofast, n (%)	1 (0.4)
Biological false positive RPR, n (%)	1 (0.4)

^a^ The number (n) of participants with results for each variable different to the total is presented. RPR: Rapid Plasma Reagin

### Reference standard and RPR results

Among the 244 participants, 112 (45.9%) were positive, 121 (49.6%) were negative and 11 (4.5%) were undetermined by the reference standard. Specifically, 2 (0.8%) had undetermined ELISA results, one (0.4%) had undetermined TPHA results, 6 (2.5%) had positive ELISA but negative TPHA results and 2 (0.8%) had positive TPHA but negative ELISA results. Among the 112 participants with positive reference standard results, 43 had a non-reactive RPR, 35 had an RPR titer <1:8 and 34 had a titer ≥ 1:8. 26 out of 234 participants (11.1%) with enough data for staging had active syphilis (Table 1).

### Performance of rapid diagnostic tests

There were 7 (2.9%) invalid results of Determine on sera, and these had to be repeated yielding negative results. There were no invalid results for Determine on CB, nor for Bioline on either type of sample. Sensitivity of the RDTs ranged from 87.5% to 99.1%. The sensitivity of Determine was significantly lower on CB than sera (87.5% vs 99.1%, p<0.001). There were no statistically significant differences in the sensitivity of Bioline on CB and sera (96.4 vs 94.6, p = 0.688). The specificity of the RDTs ranged from 96.7% to 99.2% without statistically significant differences between CB and sera. Predictive values and likelihood ratios were also similar, except for the lower values observed on CB than sera for Determine. (Table 2). Sensitivity analysis showed that RDTs had similar operating characteristics when the reference standard was TPHA alone (S2 Table), ELISA alone (S3 Table) or when the composite standard maximized its sensitivity (S4 Table).

**Table 2:** Operating characteristics of rapid diagnostic test for syphilis compared with TPHA and ELISA in people living with HIV by sample type.

Results		Bioline			Determine		
		Capillary blood	Sera	p-value^a^	Capillary blood	Sera	p-value^a^
**Rapid test results**	True positive, n	108	106	-	98	111	-
	False positive, n	2	2		1	4	
	False negative, n	4	6		14	1	
	True negative, n	119	119		120	118	
**Diagnostic accuracy**	Sensitivity, % (95% CI)	96.4	94.6	0.688^b^	87.5	99.1	**<0.001** ^b^
		(91.1–99.0)	(88.7–98.0)		(79.9–93.0)	(95.1–100)	
	Specificity, % (95% CI)	98.3	98.3	1.00^b^	99.2	96.7	0.250^b^
		(94.1–99.0)	(94.2–99.8)		(95.5–100)	(91.8–99.1)	
**Predictive values**	PPV %	98.2	98.1	0.985^c^	99.0	96.5	0.100^c^
	(95% CI)	(93.6–99.8)	(93.5–99.8)		(94.5–100)	(91.3–99.0)	
	NPV %	96.7	95.2	0.413^c^	89.6	99.2	**<0.001** ^c^
	(95% CI)	(91.9–99.1)	(89.8–98.2)		(83.1–94.2)	(95.4–100)	
**Likelihood ratios**	LR +	58.3	57.3	0.985^d^	106	30	0.052^d^
	(95% CI)	(14.8–231)	(14.5–226)		(15–746)	(11.4–78.6)	
	LR –	0.04	0.05	0.418^d^	0.13	0.01	**0.012** ^d^
	(95% CI)	(0.01–0.10)	(0.03–0.12)		(0.08–0.21)	(0.00–0.07)	

^a^ Comparing capillary blood and serum

^b^ Exact binomial test for paired data

^c^ Difference in predictive values test

^d^ Difference in diagnostic likelihood ratio test

LR+ Positive likelihood ratio, LR- Negative likelihood ratio, NPV Negative predictive values, PPV Positive predictive values.

p-values <0.05 are presented in bold

When active syphilis was used as the reference standard in PLWH without previous history of syphilis, there was a decrease in specificity to less than 86% and in the PPV of less than 37%. The likelihood ratios positive and negative were above 5 and 0.1 or lower, respectively; except for Determine on CB for which LR negative was 0.27 (Table 3).

**Table 3:** Operating characteristics of rapid diagnostic test for syphilis in people living with HIV without history of previous syphilis by sample type, using active syphilis as the reference standard.

Results		Bioline		Determine	
		Capillary blood	Sera	Capillary blood	Sera
**Rapid test results**	True positive, n	12	13	10	13
	False positive, n	24	23	19	28
	False negative, n	1	0	3	0
	True negative, n	106	107	111	102
**Diagnostic accuracy**	Sensitivity, % (95%CI)	92.3	100	76.9	100
		(64.0–99.8)	(75.3–100)	(46.2–95.0)	(75.3–100)
	Specificity, %	81.5	82.3	85.4	78.5
	(95%CI)	(73.8–87.8)	(74.6–88.4)	(78.1–91.0)	(70.4–85.2)
**Predictive values**	PPV %	33.3	36.1	34.5	31.7
	(95%CI)	(18.6–51.0)	(20.8–53.8)	(17.9–54.3)	(18.1–48.1)
	NPV %	99.1	100	95.4	100
	(95%CI)	(94.9–100)	(96.6–100)	(92.5–99.5)	(96.4–100)
**Likelihood ratios**	LR +	5	5.7	5.3	4.6
	(95%CI)	(3.4–7.4)	(3.9–8.2)	(3.2–8.8)	(3.3–6.5)
	LR –	0.094	0	0.27	0
	(95%CI)	(0.014–0.621)	(-)	(0.010–0.731)	(-)

LR+ Positive likelihood ratio, LR- Negative likelihood ratio, NPV Negative predictive values, PPV Positive predictive values.

### Sources of heterogeneity

**Fig 2:**
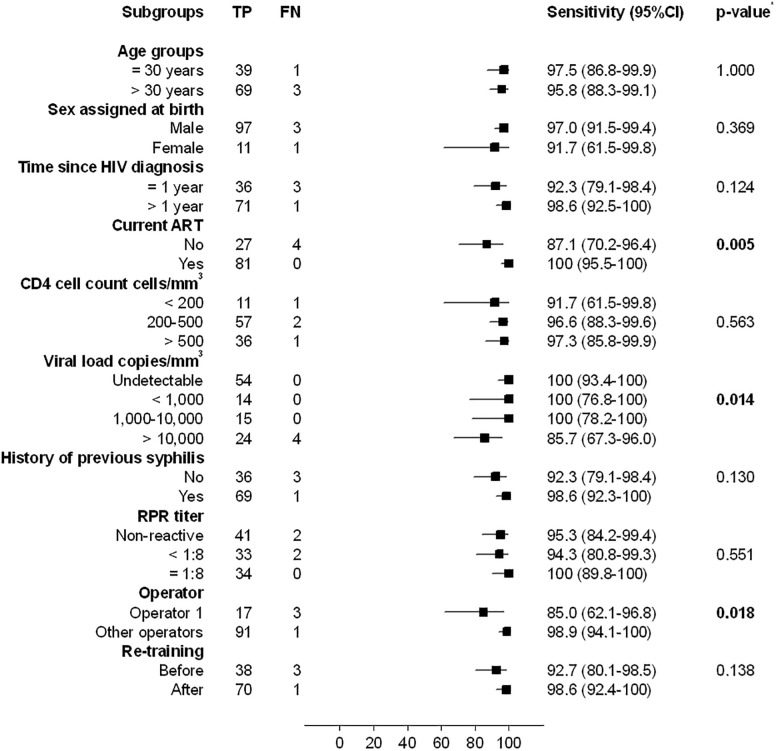
Sensitivity of Bioline on capillary blood stratified by demographic, clinical and technical factors. * Fisher’s exact test.

Stratified analysis revealed that the sensitivity of Bioline using CB was lower in PLWH not receiving ART (87.1% vs 100%, p = 0.005), with most recent viral load >10,000 copies/mm^3^ (85.7% vs 100, p = 0.014) and when the tests were performed by operator 1 (85% vs 98.9%, p = 0.018) (Fig 2). However, when RDTs performed by operator 1 were excluded, the observed differences were attenuated and were no longer statistically significant (S1 Fig). Nevertheless, stratified analysis of Bioline using sera showed a lower sensitivity in PLWH not receiving ART (87.1% vs 97.5%, p = 0.049) and with detectable viral loads (p = 0.008) (S2 Fig).

**Fig 3:**
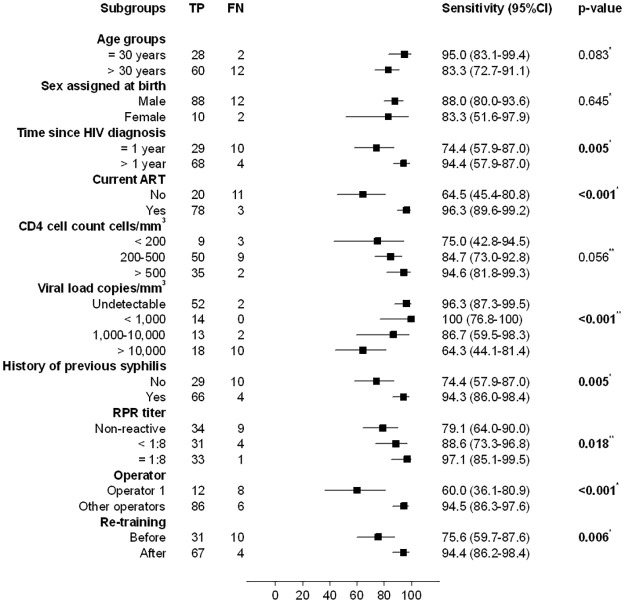
Sensitivity of determine on capillary blood stratified by demographic, clinical and technical factors. * Fisher’s exact test ** Cuzick test for trend.

The sensitivity of Determine using CB was lower in PLWH diagnosed ≤ 1 year before enrolment (74.4% vs 94.4%, p = 0.005), not receiving ART (64.5% vs 94.4%, p<0.001), with higher viral loads (p for trend <0.001), without previous history of syphilis (74.4% vs 94.3%, p = 0.005), lower RPR titers (p for trend = 0.018), and when the tests were performed by operator 1 (60% vs 94.5%, p<0.001). The re-training of operators increased the sensitivity of Determine on CB from 75.6% (59.5%-87.6%) to 94.4% (86.2%-98.4%) (p = 0.006) (Fig 3). However, when the results of RDTs performed by operator 1 were excluded, some of the differences were attenuated but remained significant (S3 Fig), e.g. while the sensitivity of Determine on CB remained lower in PLWH diagnosed ≤ 1 year before enrolment, not receiving ART and with higher viral loads, the sensitivity was not significantly lower with lower RPR titers or during the period before re-training. Nevertheless, none of the differences were statistically significant when the analysis was performed for Determine on sera (S4 Fig).

Specificity of RDTs was similar in the stratified analysis by age, sex, time since HIV diagnosis, ART, CD4 cells count, viral load, history of syphilis, RPR titers, operator, and re-training (S5–S8 Figs).

### Quality control, compliance and adverse events

The concordance between the three blinded evaluators of RDTs on sera was almost perfect for both Determine (κ = 0.98 95% CI 0.97–1.00) and Bioline (κ = 0.99 95% CI 0.98–1.00). The agreement between the 20 tests (10 Bioline and 10 Determine) stored under field conditions in the clinic and the tests stored in the reference laboratory was 100%. In most participants (238, 97.5%) finger prick and venipuncture were performed on the same date. However, in 5 cases (2%) the venipuncture was performed one day after the finger prick and in 1 case (0.4%) it was performed 8 days later. The median time between venipuncture and processing of reference tests in thawed sera samples was 31 days (range 3–59). There were no severe adverse events associated with finger prick or venipuncture. However, we had to perform a second finger prick in 13 (5.3%) participants due to insufficient sample for both RDTs.

## Discussion

Our results provide evidence that the diagnostic performance of the RDTs in PLWH is excellent when compared to TPHA and ELISA. Particularly, the results support the use of Bioline in either CB or sera and Determine on sera. These results are important because RDTs has been widely implemented in Colombia [34] and other low and middle income countries [13] for the screening of syphilis in pregnant women independently of HIV status and the current Colombian HIV care guidelines recommends their use in the HIV care programs [16]. We also identified several sources of heterogeneity in the sensitivity of RDTs that may explain the highly heterogeneous results of previous field validation studies [10, 11] with prediction intervals derived from random effects models for meta-analysis ranging from 57% to 96% [11]. In this study, operational factors were a major source of heterogeneity in the sensitivity of RDTs. While Determine had the highest sensitivity when performed using sera it had the lowest sensitivity using CB, particularly when performed by Operator 1. Nonetheless, its sensitivity increased after the re-training which underscores the importance of monitoring the individual performance of operators and the scheduling of additional training sessions during the implementation of RDTs. The lower sensitivity of RDTs, particularly Determine, when performed by Operator 1 reflect the challenges of implementing RDTs requiring larger volumes of CB (50 μL for Determine versus 20 μL for Bioline) in busy clinical settings where the same sample is used for several rapid tests, as may be the case of point-of-care testing for sexually transmitted infections [35]. In our study, Operator 1 also had to perform rapid CD4 and HIV p24 antigen tests. In other settings, testing for other infectious diseases may be required, potentially leading to false negative syphilis results if sample volume is compromised. Therefore, considering that capillary blood is the target sample for point-of-care testing in resource-sparse settings [36], our results suggest that the required sample volume should be taken into consideration during the selection of RDTs for syphilis screening programs when multiple tests may be performed at the same time. Development of rapid testing devices for the screening of several infectious diseases at the same time and with the same sample might also overcome this potential limitation [37].

We also identified biological sources of heterogeneity in the sensitivity of RDTs. As previously reported [17, 18], sensitivity of RDTs was higher when non-treponemal tests were reactive. Furthermore, we found that the sensitivity of Bioline on CB was more than 90% for detection of active syphilis among those without previous history of syphilis and therefore may be particularly useful for early identification and treatment of infectious cases, with greater transmission potential in the population. We also identified that factors associated with the immune status of the host (no use of ART and higher viral load) were associated with lower sensitivity of RDTs, independently of diagnostic test, sample type and operator. In a study carried out in Mozambique between 2003 and 2004, Montoya *et al* [17] reported an association between HIV co-infection and risk of false negative results in RDTs. However, the HIV cases were detected through counseling and testing and were likely not receiving ART at the time of syphilis testing. In contrast, the study by Causer *et al* [14], was carried out in Australia and used archive specimens that were more likely to have been from PLWH receiving ART. In agreement with the results by Causer *et al*, we did not find statistically significant differences in the sensitivity of RDTs by CD4 counts. These results may be related to impaired production of specific antibodies against *T*. *pallidum* induced by active HIV replication as described for other infectious diseases [38].

Our study had some limitations. First, while we reached the sample size to validate the accuracy of RDTs to detect treponemal antibodies, our sample included few cases of active syphilis and we were unable to explore the effect of syphilis stage on the sensitivity of the RDTs. However, our diagnostic performance estimates using active syphilis as reference standard followed a similar pattern to those observed with the composite treponemal reference standard. Nevertheless, the confidence intervals are wide and previous studies have shown that serologic tests for syphilis have lower sensitivities during primary syphilis [23] and therefore our results may overestimate the sensitivity of the RDTs since we only included one of those cases. Furthermore, operators were not masked to clinical data or the results of the other RDT when performed in capillary blood and we did not randomize the order of the tests, thus the interpretation of RDTs results in capillary blood were not independent and their sensitivity may have been overestimated [39]. Another limitation was that Operator 1, who had the lowest sensitivity of RDTs, mainly performed tests in newly diagpnosed HIV cases and in those who had been previously lost to follow up from the HIV care program, potentially confounding the estimation of sensitivity of RDTs in PLWH not receiving ART and with higher viral loads. However, restricted analysis excluding tests performed by Operator 1, and the results from sera, showed similar, though attenuated, results, which suggests a biological basis for the lower performance in these groups. Finally, the use of a composite algorithm may affect the estimation of test accuracy [30]. However, the consistent results from sensitivity analysis with alternative reference tests suggest this does not seem to be a major concern. Our study had several further strengths including the quality assurance system, the identification of concordance among readers of RDTs in sera, and the masking of sera samples for RDTs and reference standards to minimize observer and classification bias. In addition, participants were recruited consecutively in several clinics likely to reflect the target population of RDTs for the diagnosis of syphilis in PLWH.

In conclusion, our results support the use of Determine and Bioline RDTs for the screening of syphilis in PLWHA, the former on sera and the latter on both sera and CB. Operational factors must be considered during the implementation of RDTs to sustain test performance; hence, RDTs with less arduous blood volume requirements should be preferred for point-of-care testing. Sources of heterogeneity should be considered during the interpretation of test results. RDTs have the potential to help to close the gap in syphilis screening coverage in resource-sparse settings, reducing the morbidity and transmission through shorter time to treatment. Further studies are required to assess the performance of RDTs to screen for active syphilis, the impact, and cost-effectiveness of a test-and-treat strategy using an RDT based screening algorithm in PLWH.

## Supporting information

S1 Table STARD checklist. (PDF)

S2 Table Operating characteristics of rapid diagnostic test for syphilis in people living with HIV by sample type, using TPHA as the reference standard. (PDF)

S3 Table Operating characteristics of rapid diagnostic test for syphilis in people living with HIV by sample type, using ELISA as the reference standard. (PDF)

S4 Table Operating characteristics of rapid diagnostic test for syphilis in people living with HIV by sample type, using a composite result maximizing sensitivity as the reference standard. (PDF)

S1 Fig Sensitivity of Bioline in capillary blood stratified by demographic, clinical and technical factors including test performed by operators other than operator 1. (PDF)

S2 Fig Sensitivity of Bioline on sera stratified by demographic, clinical and technical factors. (PDF)

S3 Fig Sensitivity of determine on capillary blood stratified by demographic, clinical and technical factors including test performed by operators other than operator 1. (PDF)

S4 Fig Sensitivity of determine on sera stratified by demographic, clinical and technical factors. (PDF)

S5 Fig Specificity of Bioline on sera stratified by demographic, clinical and technical factors. (PDF)

S6 Fig Specificity of Determine on capillary blood stratified by demographic, clinical and technical factors. (PDF)

S7 Fig Specificity of Bioline on sera stratified by demographic, clinical and technical factors. (PDF)

S8 Fig Specificity of determine on sera stratified by demographic, clinical and technical factors. (PDF)

## Abbreviations

95% CI: 95% confidence interval
ART: Antiretroviral therapy
CB: Capillary blood
CIDEIM: Centro Internacional de Entrenamiento e Investigaciones Médicas [International center for medical training and research]
eCRF: Electronic case report file
ELISA: Enzyme linked immunosorbent assay
HIV: Human immunodeficiency virus
INVIMA: Institutio Nacional de Vigilancia de Medicamentos y Alimentos [National Institute of food and drugs surveillance
LR: Likelihood ratios
NTT: Non treponemal test
PLWH: People living with HIV
RDTs: Rapid diagnostic tests
STARD: Standard for the Reporting of Diagnostic Accuracy Studies
TPHA: *Treponema pallidum* haemagglutination assay
TT: Treponemal test
