# Correction: Diagnostic performance of two rapid tests for syphilis screening in people living with HIV in Cali, Colombia

**DOI:** 10.1371/journal.pone.0320868

**Published:** 2025-03-19

**Authors:** Jonny Alejandro García Luna, Nelson Romero-Rosas, Sebastian Alejandro Silva Peña, Oscar Javier Oviedo Sarmiento, Ximena Galindo Orrego, William Lenis Quintero, Luisa Consuelo Rubiano, Ernesto Martínez Buitrago, Lyda Osorio, Juan Carlos Salazar, Adrian D. Smith, Neal Alexander

The seventh author’s name is listed incorrectly. The correct name is: Luisa Consuelo Rubiano.
