# Supplementary material for: Diagnostic performance of two rapid tests for syphilis screening in people living with HIV in Cali, Colombia
Source: PLoS One. 2023 Mar 9;18(3):e0282492. doi: 10.1371/journal.pone.0282492 (PMC9997911; doi:10.1371/journal.pone.0282492)
Supplement: S1 Table — (PDF) [file pone.0282492.s001.pdf]

S1 Table. STARD Checklist

	Section & Topic	No	Item	Reported on page #
	**TITLE OR ABSTRACT**			
		**1**	Identification as a study of diagnostic accuracy using at least one measure of accuracy (such as sensitivity, specificity, predictive values, or AUC)	1
	**ABSTRACT**			
		**2**	Structured summary of study design, methods, results, and conclusions (for specific guidance, see STARD for Abstracts)	2
	**INTRODUCTION**			
		**3**	Scientific and clinical background, including the intended use and clinical role of the index test	Second and third paragraphs of the introduction
		**4**	Study objectives and hypotheses	Last sentence of the introduction
	**METHODS**			
	Study design	**5**	Whether data collection was planned before the index test and reference standard were performed (prospective study) or after (retrospective study)	Study design, population and setting subheading of the methods section
	Participants	**6**	Eligibility criteria	Study design, population and setting subheading of the methods section
		**7**	On what basis potentially eligible participants were identified (such as symptoms, results from previous tests, inclusion in registry)	Study design, population and setting subheading of the methods section
		**8**	Where and when potentially eligible participants were identified (setting, location and dates)	Study design, population and setting subheading of the methods section
		**9**	Whether participants formed a consecutive, random or convenience series	Study design, population and setting subheading of the methods section
	Test methods	**10a**	Index test, in sufficient detail to allow replication	Index and reference tests subheading of the methods section
		**10b**	Reference standard, in sufficient detail to allow replication	Index and reference tests subheading of the methods section
		**11**	Rationale for choosing the reference standard (if alternatives exist)	Index and reference tests subheading of the methods section
		**12a**	Definition of and rationale for test positivity cut-offs or result categories<br>of the index test, distinguishing pre-specified from exploratory	Index and reference tests subheading of the methods section
		**12b**	Definition of and rationale for test positivity cut-offs or result categories of the reference standard, distinguishing pre-specified from exploratory	Index and reference tests subheading of the methods section
		**13a**	Whether clinical information and reference standard results were available to the performers/readers of the index test	Index and reference tests subheading of the methods section
		**13b**	Whether clinical information and index test results were available to the assessors of the reference standard	Reference tests and serum RDTs subheading of the methods section
	Analysis	**14**	Methods for estimating or comparing measures of diagnostic accuracy	Statistical analysis subheading of the methods section

		**15**	How indeterminate index test or reference standard results were handled	Statistical analysis subheading of the methods section
		**16**	How missing data on the index test and reference standard were handled	There were not missing data
		**17**	Any analyses of variability in diagnostic accuracy, distinguishing pre-specified from exploratory	Statistical analysis subheading of the methods section
		**18**	Intended sample size and how it was determined	Sample size subheading of the methods section
	**RESULTS**			
	Participants	**19**	Flow of participants, using a diagram	Fig 1
		**20**	Baseline demographic and clinical characteristics of participants	Table 1
		**21a**	Distribution of severity of disease in those with the target condition	Table 1
		**21b**	Distribution of alternative diagnoses in those without the target condition	Not applicable. Syphilis tests were intended as screening tests
		**22**	Time interval and any clinical interventions between index test and reference standard	Compliance, quality control and adverse events subheadings of the results section
	Test results	**23**	Cross tabulation of the index test results (or their distribution)<br>by the results of the reference standard	Table 2
		**24**	Estimates of diagnostic accuracy and their precision (such as 95% confidence intervals)	Table 2
		**25**	Any adverse events from performing the index test or the reference standard	Compliance, quality control and adverse events subheadings of the results section
	**DISCUSSION**			
		**26**	Study limitations, including sources of potential bias, statistical uncertainty, and generalisability	Fourth paragraph of the discussion
		**27**	Implications for practice, including the intended use and clinical role of the index test	First, second and last paragraph od the discussion
	**OTHER INFORMATION**			
		**28**	Registration number and name of registry	Not available
		**29**	Where the full study protocol can be accessed	Not available
		**30**	Sources of funding and other support; role of funders	Funding section
