# Supplementary material for: Diagnostic performance of two rapid tests for syphilis screening in people living with HIV in Cali, Colombia
Source: PLoS One. 2023 Mar 9;18(3):e0282492. doi: 10.1371/journal.pone.0282492 (PMC9997911; doi:10.1371/journal.pone.0282492)
Supplement: S2 Table — (PDF) [file pone.0282492.s002.pdf]

S2 Table. Operating characteristics of rapid diagnostic test for syphilis in people living with HIV by sample type, using TPHA as the reference standard.

**Results**		**Bioline**		**Determine**	
		Capillary blood	Sera	Capillary blood	Sera
**Rapid test results**	True positive, n	109	113	100	114
	False positive, n	7	11	8	3
	False negative, n	5	1	14	5
	True negative, n	122	118	121	120
**Diagnostic accuracy**	Sensitivity, % (95%CI)	95.6 (90.1-98.6)	99.1 (95.2-100)	87.7 (80.3-93.1)	95.8 (90.5-98.6)
	Specificity, % (95%CI)	94.6 (89.1-97.8)	91.5 (85.3-95.7)	93.8 (88.1-97.3)	97.6 (93.0-99.5)
**Predictive values**	PPV % (95%CI)	94.0 (88.0-97.5)	91.1 (84.7-95.5)	92.6 (85.9-96.7)	97.4 (92.7-99.5)
	NPV % (95%CI)	96.1 (91.1-98.7)	99.2 (95.4-100)	89.6 (83.2-94.2)	96.0 (90.9-98.7)
**Likelihood ratios**	LR + (95%CI)	17.6 (8.6-36.3)	11.6 (6.6-20.5)	14.1 (7.2-27.8)	39.3 (12.8-120)
	LR – (95%CI)	0.05 (0.02-0.11)	0.01 (0.00-0.07)	0.13 (0.08-0.21)	0.04 (0.02-0.10)

LR+ Positive likelihood ratio, LR- Negative likelihood ratio, NPV Negative predictive values, PPV Positive predictive values.
