# Supplementary material for: Diagnostic performance of two rapid tests for syphilis screening in people living with HIV in Cali, Colombia
Source: PLoS One. 2023 Mar 9;18(3):e0282492. doi: 10.1371/journal.pone.0282492 (PMC9997911; doi:10.1371/journal.pone.0282492)
Supplement: S3 Table — (PDF) [file pone.0282492.s003.pdf]

S3 Table. Operating characteristics of rapid diagnostic test for syphilis in people living with HIV by sample type, using ELISA as the reference standard

**Results**		**Bioline**		**Determine**	
		Capillary blood	Sera	Capillary blood	Sera
**Rapid test results**	True positive, n	114	112	104	117
	False positive, n	3	3	3	6
	False negative, n	5	7	15	2
	True negative, n	7	120	120	117
**Diagnostic accuracy**	Sensitivity, % (95%CI)	95.8 (90.5-98.6)	94.1 (88.3-97.6)	87.4 (80.1-92.8)	98.3 (94.1-99.8)
	Specificity, % (95%CI)	97.6 (93.0-99.5)	97.6 (93.0-99.5)	97.6 (93.0-99.5)	95.1 (89.7-98.2)
**Predictive values**	PPV % (95%CI)	97.4 (92.7-99.5)	97.4 (92.6-99.5)	97.2 (92.0-99.4)	95.1 (89.7-98.2)
	NPV % (95%CI)	96.0 (90.9-98.7)	94.5 (89.0-97.8)	88.9 (82.3-93.6)	98.3 (94.1-99.8)
**Likelihood ratios**	LR + (95%CI)	39.3 (12.8-120)	38.6 (12.6-118)	35.8 (11.7-110)	20.2 (9.23-44)
	LR – (95%CI)	0.04 (0.02-0.10)	0.06 (0.03-0.12)	0.13 (0.08-0.21)	0.02 (0.00-0.07)

LR+ Positive likelihood ratio, LR- Negative likelihood ratio, NPV Negative predictive values, PPV Positive predictive values.
