# Supplementary material for: Diagnostic performance of two rapid tests for syphilis screening in people living with HIV in Cali, Colombia
Source: PLoS One. 2023 Mar 9;18(3):e0282492. doi: 10.1371/journal.pone.0282492 (PMC9997911; doi:10.1371/journal.pone.0282492)
Supplement: S4 Table — (PDF) [file pone.0282492.s004.pdf]

S4 Table. Operating characteristics of rapid diagnostic test for syphilis in people living with HIV by sample type, using a composite result maximising sensitivity as the reference standard

**Results**		**Bioline**		**Determine**	
		Capillary blood	Sera	Capillary blood	Sera
**Rapid test results**	True positive, n	115	113	106	119
	False positive, n	2	2	1	4
	False negative, n	6	8	15	2
	True negative, n	119	119	120	117
**Diagnostic accuracy**	Sensitivity, % (95%CI)	95.0 (89.5-98.2)	93.4 (87.4-97.1)	87.6 (80.4-92.9)	98.3 (94.2-99.8)
	Specificity, % (95%CI)	98.3 (94.2-99.8)	98.3 (94.2-99.8)	99.2 (95.5-100)	96.7 (91.8-99.1)
**Predictive values**	PPV % (95%CI)	98.3 (93.9-99.8)	98.3 (93.9-99.8)	99.1 (94.9-100)	96.7 (91.9-99.1)
	NPV % (95%CI)	95.2 (89.8-98.2)	93.7 (88.0-97.2)	88.9 (82.3-93.6)	98.3 (94.1-99.8)
**Likelihood ratios**	LR + (95%CI)	57.5 (14.5-227)	56.5 (14.3-224)	106 (15.0-747.0)	29.8 (11.3-78)
	LR – (95%CI)	0.05 (0.02-0.11)	0.07 (0.03-0.13)	29.8 (11.3-78.0)	0.02 (0.00-0.07)

LR+ Positive likelihood ratio, LR- Negative likelihood ratio, NPV Negative predictive values, PPV Positive predictive values.
