# Supplementary material for: Diagnostic performance of two rapid tests for syphilis screening in people living with HIV in Cali, Colombia
Source: PLoS One. 2023 Mar 9;18(3):e0282492. doi: 10.1371/journal.pone.0282492 (PMC9997911; doi:10.1371/journal.pone.0282492)
Supplement: S1 Fig — (PDF) [file pone.0282492.s005.pdf]

Subgroups	TP	FN	Sensitivity (95%CI)	p-value*
**Age groups**				
≤ 30 years	33	0	100 (89.4-100)	1.000
> 30 years	58	1	98.3 (90.9-100)	
**Sex assigned at birth**				
Male	82	0	100 (95.6-100)	0.109
Female	9	1	90.0 (55.5-99.7)	
**Time since HIV diagnosis**				
≤ 1 year	23	1	95.8 (78.9-99.9)	0.264
> 1 year	67	0	100 (94.6-100)	
**Current ART**				
No	13	1	92.9 (66.1-99.8)	0.152
Yes	78	0	100 (95.4-100)	
**CD4 cell count cells/mm^3**				
< 200	8	0	100 (63.1-100)	
200-500	47	0	100 (92.5-100)	0.466
> 500	32	1	97.0 (84.2-99.9)	
**Viral load copies/mm^3**				
Undetectable	53	0	100 (93.3-100)	
< 1,000	13	0	100 (75.3-100)	0.418
1,000-10,000	10	0	100 (69.2-100)	
> 10,000	14	1	93.3 (68.1-99.8)	
**History of previous syphilis**				
No	24	0	100 (85.8-100)	1.000
Yes	65	1	98.5 (91.8-100)	
**RPR titer**				
Non-reactive	35	1	97.2 (85.5-99.9)	
< 1:8	26	0	100 (86.8-100)	1.000
≥ 1:8	30	0	100 (88.6-100)	
**Re-training**				
Before	26	1	96.3 (81.0-99.9)	0.293
After	65	0	100 (94.5-100)	

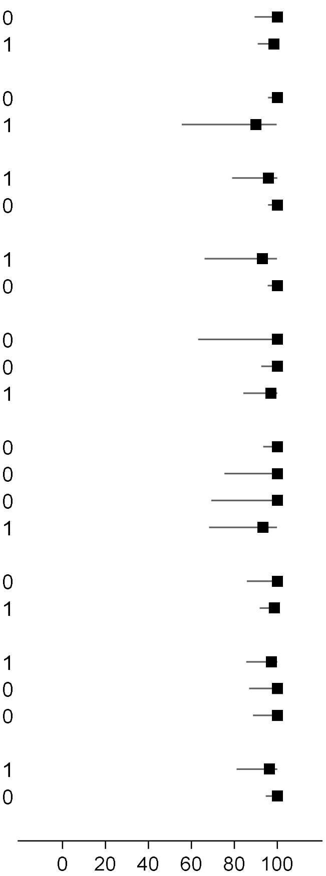

Sensitivity (%) of SD Bioline in capillary blood compared to TPHA and ELISA in sera from PLWH

*Fisher's exact test

S1 Fig. Sensitivity of Bioline on capillary blood stratified by demographic, clinical and technical factors including test performed by operators other than operator 1
