# Supplementary material for: Diagnostic performance of two rapid tests for syphilis screening in people living with HIV in Cali, Colombia
Source: PLoS One. 2023 Mar 9;18(3):e0282492. doi: 10.1371/journal.pone.0282492 (PMC9997911; doi:10.1371/journal.pone.0282492)
Supplement: S2 Fig — (PDF) [file pone.0282492.s006.pdf]

Subgroups	TP	FN	Sensitivity (95%CI)	p-value*
**Age groups**				
≤ 30 years	37	3	92.5 (79.6-98.4)	0.665
> 30 years	69	3	95.8 (88.3-99.1)	
**Sex assigned at birth**				
Male	95	5	95.0 (88.7-98.4)	0.502
Female	11	1	91.7 (61.5-99.8)	
**Time since HIV diagnosis**				
≤ 1 year	36	3	92.3 (79.1-98.4)	0.422
> 1 year	69	3	95.8 (88.3-99.1)	
**Current ART**				
No	27	4	87.1 (70.2-96.4)	**0.049**
Yes	79	2	97.5 (91.4-99.7)	
**CD4 cell count cells/mm^3**				
< 200	12	0	100 (73.5-100)	
200-500	55	4	93.2 (83.5-98.1)	1.000
> 500	35	2	94.6 (81.8-99.3)	
**Viral load copies/mm^3**				
Undetectable	54	0	100 (93.4-100)	
< 1,000	12	2	85.7 (57.2-98.2)	**0.008**
1,000-10,000	15	0	100 (78.2-100)	
> 10,000	24	4	85.7 (67.3-96.0)	
**History of previous syphilis**				
No	35	4	89.7 (75.8-97.1)	0.184
Yes	68	2	97.1 (90.1-99.7)	
**RPR titer**				
Non-reactive	40	3	93 (80.9-98.5)	
< 1:8	32	3	91.4 (76.9-98.2)	0.314
≥ 1:8	34	0	100 (89.8-100)	

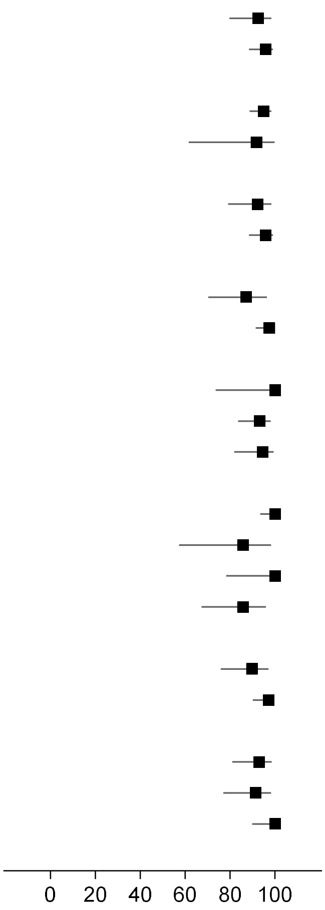

Sensitivity (%) of SD Bioline in sera compared to TPHA and ELISA in sera from PLWH

*Fisher's exact test

S2 Fig. Sensitivity of Bioline on sera stratified by demographic, clinical and technical factors.
