# Supplementary material for: Diagnostic performance of two rapid tests for syphilis screening in people living with HIV in Cali, Colombia
Source: PLoS One. 2023 Mar 9;18(3):e0282492. doi: 10.1371/journal.pone.0282492 (PMC9997911; doi:10.1371/journal.pone.0282492)
Supplement: S3 Fig — (PDF) [file pone.0282492.s007.pdf]

Subgroups	TP	FN	Sensitivity (95%CI)	p-value
**Age groups**				
≤ 30 years	32	1	97.0 (84.2-99.9)	0.414*
> 30 years	54	5	91.5 (81.3-97.2)	
**Sex assigned at birth**				
Male	77	5	93.9 (86.3-98.0)	0.418*
Female	9	1	90.0 (55.5-99.7)	
**Time since HIV diagnosis**				
≤ 1 year	20	4	83.3 (62.6-95.3)	**0.040***
> 1 year	65	2	97 (89.6-99.6)	
**Current ART**				
No	10	4	71.4 (41.9-91.6)	**0.004***
Yes	76	2	97.4 (91.0-99.7)	
**CD4 cell count cells/mm^3**				
< 200	8	0	100 (63.1-100)	
200-500	42	5	89.4 (76.9-96.5)	0.537**
> 500	32	1	97.0 (84.2-99.9)	
**Viral load copies/mm^3**				
Undetectable	52	1	98.1 (89.9-100)	
< 1,000	13	0	100 (75.3-100)	**0.001****
1,000-10,000	9	1	90.0 (55.5-99.7)	
> 10,000	11	4	73.3 (44.9-92.2)	
**History of previous syphilis**				
No	21	3	87.5 (67.6-97.3)	0.336*
Yes	63	3	95.5 (87.3-99.1)	
**RPR titer**				
Non-reactive	31	5	86.1 (70.5-95.3)	
< 1:8	26	0	100 (86.8-100)	**0.073****
≥ 1:8	29	1	96.7 (83.3-99.4)	
**Re-training**				
Before	24	3	88.9 (70.8-97.6)	0.354*
After	62	3	95.4 (87.1-99.0)	

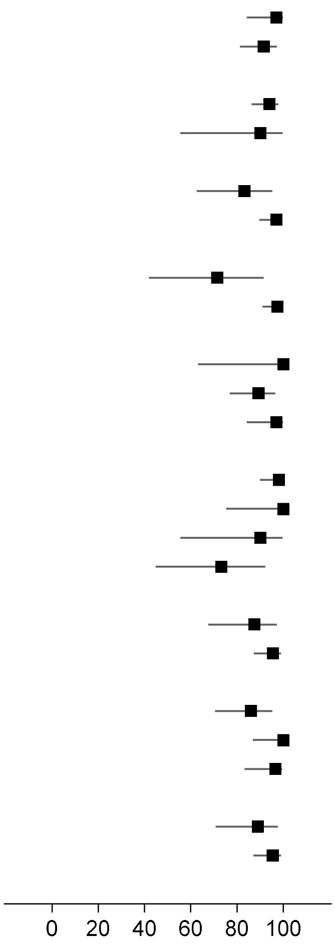

Sensitivity (%) of Determine in capillary blood compared to TPHA and ELISA in sera from PLWH

*Fisher's exact test

S3 Fig. Sensitivity of Determine on capillary blood stratified by demographic, clinical and technical factors including test performed by operators other than operator 1
