# Supplementary material for: Diagnostic performance of two rapid tests for syphilis screening in people living with HIV in Cali, Colombia
Source: PLoS One. 2023 Mar 9;18(3):e0282492. doi: 10.1371/journal.pone.0282492 (PMC9997911; doi:10.1371/journal.pone.0282492)
Supplement: S4 Fig — (PDF) [file pone.0282492.s008.pdf]

| Subgroups | TP | FN | Sensitivity (95%CI) | p-value* |
|---|---|---|---|---|
| **Age groups** | | | | |
| ≤ 30 years | 40 | 0 | 100 (91.2-100) | 1.000 |
| > 30 years | 71 | 1 | 98.6 (92.5-100) | |
| **Sex assigned at birth** | | | | |
| Male | 100 | 0 | 100 (96.4-100) | 0.107 |
| Female | 11 | 1 | 91.7 (61.5-99.8) | |
| **Time since HIV diagnosis** | | | | |
| ≤ 1 year | 39 | 0 | 100 (91.0-100) | 1.000 |
| > 1 year | 71 | 1 | 98.6 (92.5-100) | |
| **Current ART** | | | | |
| No | 31 | 0 | 100 (88.8-100) | 1.000 |
| Yes | 80 | 1 | 98.8 (93.3-100) | |
| **CD4 cell count cells/mm$^3$** | | | | |
| < 200 | 12 | 0 | 100 (73.5-100) | |
| 200-500 | 58 | 1 | 98.3 (90.9-100) | 1.000 |
| > 500 | 37 | 0 | 100 (90.5-100) | |
| **Viral load copies/mm$^3$** | | | | |
| Undetectable | 53 | 1 | 98.1 (90.1-100) | |
| < 1,000 | 14 | 0 | 100 (76.8-100) | 1.000 |
| 1,000-10,000 | 15 | 0 | 100 (78.2-100) | |
| > 10,000 | 28 | 0 | 100 (87.7-100) | |
| **History of previous syphilis** | | | | |
| No | 39 | 0 | 100 (91.0-100) | 1.000 |
| Yes | 69 | 1 | 98.6 (92.3-100) | |
| **RPR titer** | | | | |
| Non-reactive | 43 | 0 | 100 (91.8-100) | |
| < 1:8 | 34 | 1 | 97.1 (85.1-99.9) | 0.616 |
| ≥ 1:8 | 34 | 0 | 100 (89.8-100) | |

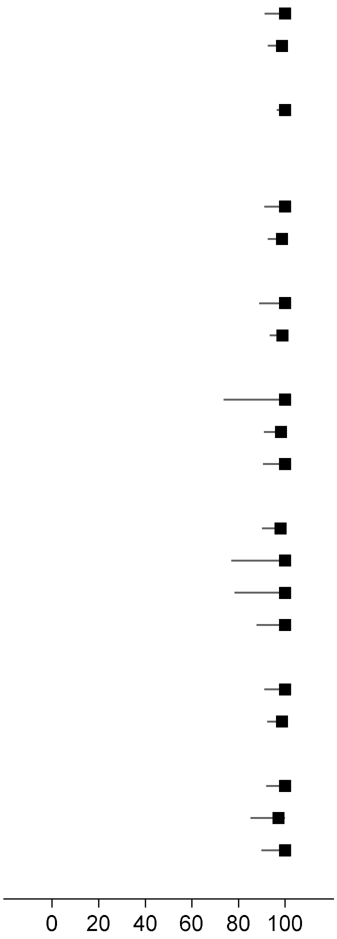


**Sensitivity (%) of Determine in sera compared to TPHA and ELISA in sera from PLWH**

*Fisher's exact test

**S4 Fig. Sensitivity of Determine on sera stratified by demographic, clinical and technical factors.**
