# Supplementary material for: Diagnostic performance of two rapid tests for syphilis screening in people living with HIV in Cali, Colombia
Source: PLoS One. 2023 Mar 9;18(3):e0282492. doi: 10.1371/journal.pone.0282492 (PMC9997911; doi:10.1371/journal.pone.0282492)
Supplement: S5 Fig — (PDF) [file pone.0282492.s009.pdf]

Subgroups	TN	FP	Specificity (95%CI)	p-value*
**Age groups**				
≤ 30 years	39	0	100 (91.0-100)	1.000
> 30 years	80	2	97.6 (91.5-99.7)	
**Sex assigned at birth**				
Male	78	2	97.5 (91.3-99.7)	0.548
Female	41	0	100 (91.4-100)	
**Time since HIV diagnosis**				
≤ 1 year	41	1	97.6 (87.4-99.9)	1.000
> 1 year	77	1	98.7 (93.1-100)	
**Current ART**				
No	44	0	100 (92.0-100)	0.533
Yes	75	2	97.4 (90.9-99.7)	
**CD4 cell count cells/mm^3**				
< 200	21	0	100 (83.9-100)	
200-500	58	2	96.7 (88.5-99.6)	0.669
> 500	40	0	100 (91.2-100)	
**Viral load copies/mm^3**				
Undetectable	66	1	98.5 (92.0-100)	
< 1,000	10	0	100 (69.2-100)	0.391
1,000-10,000	10	1	90.9 (58.7-99.8)	
> 10,000	33	0	100 (89.4-100)	
**History of previous syphilis**				
No	104	1	99 (94.8-100)	0.137
Yes	7	1	87.5 (47.3-99.7)	
**RPR titer**				
Non-reactive	118	2	98.3 (94.1-99.8)	
< 1:8	1	0	100 (2.5-100)	1.000
≥ 1:8	0	0	-	
**Operator**				
Operator 1	30	0	100 (89.4-100)	1.000
Other operators	89	2	97.8 (92.3-99.7)	
**Re-training**				
Before	33	0	100 (89.4-100)	1.000
After	86	2	97.7 (92.0-100)	

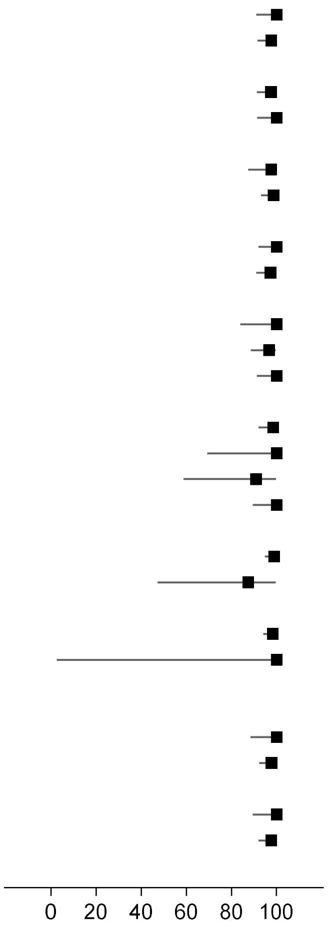

Specificity (%) of SD Bioline in capillary blood compared to TPHA and ELISA in sera from PLWH

Fisher's exact test

S5 Fig. Specificity of Bioline on capillary blood stratified by demographic, clinical and technical factors.
