# Supplementary material for: Diagnostic performance of two rapid tests for syphilis screening in people living with HIV in Cali, Colombia
Source: PLoS One. 2023 Mar 9;18(3):e0282492. doi: 10.1371/journal.pone.0282492 (PMC9997911; doi:10.1371/journal.pone.0282492)
Supplement: S6 Fig — (PDF) [file pone.0282492.s010.pdf]

Subgroups	TN	FP	Specificity (95%CI)	p-value*
**Age groups**				
≤ 30 years	39	0	100 (91.0-100)	1.000
> 30 years	81	1	98.8 (93.4-100)	
**Sex assigned at birth**				
Male	80	0	100 (95.5-100)	0.339
Female	40	1	97.6 (87.1-99.9)	
**Time since HIV diagnosis**				
≤ 1 year	42	0	100 (91.6-100)	1.000
> 1 year	77	1	98.7 (93.1-100)	
**Current ART**				
No	44	0	100 (92.0-100)	0.533
Yes	76	1	98.7 (93.0-100)	
**CD4 cell count cells/mm^3**				
< 200	20	1	95.2 (76.1-99.9)	
200-500	60	0	100 (94.0-100)	0.174
> 500	40	0	100 (91.2-100)	
**Viral load copies/mm^3**				
Undetectable	66	1	96.3 (87.3-99.5)	
< 1,000	10	0	100 (69.2-100)	1.000
1,000-10,000	11	0	100 (71.5-100)	
> 10,000	33	0	100 (89.4-100)	
**History of previous syphilis**				
No	104	1	99.0 (94.8-100)	1.000
Yes	8	0	100 (63.1-100)	
**RPR titer**				
Non-reactive	119	1	99.2 (95.4-100)	
< 1:8	1	0	100 (2.5-100)	1.000
≥ 1:8	0	0	-	
**Operator**				
Operator 1	30	0	100 (88.4-100)	1.000
Other operators	90	1	98.9 (94.0-100)	
**Re-training**				
Before	33	0	100 (89.4-100)	1.000
After	87	1	98.9 (93.8-100)	

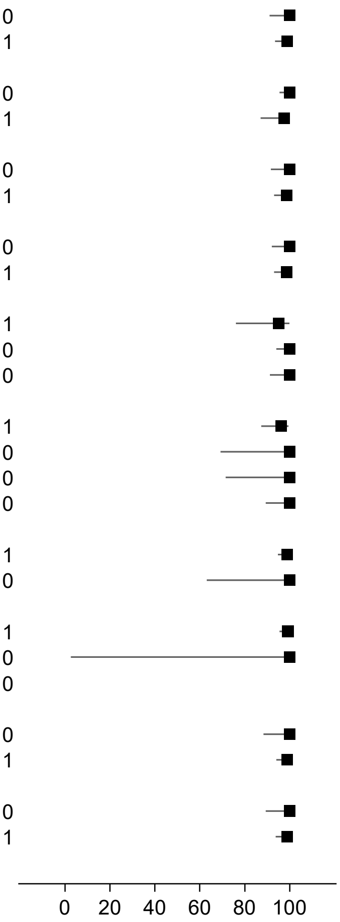

Specificity (%) of Determine in capillary blood compared to TPHA and ELISA in sera from PLWH

*Fisher's exact test

S6 Fig. Specificity of Determine on capillary blood stratified by demographic, clinical and technical factors.
