# Supplementary material for: Diagnostic performance of two rapid tests for syphilis screening in people living with HIV in Cali, Colombia
Source: PLoS One. 2023 Mar 9;18(3):e0282492. doi: 10.1371/journal.pone.0282492 (PMC9997911; doi:10.1371/journal.pone.0282492)
Supplement: S7 Fig — (PDF) [file pone.0282492.s011.pdf]

Subgroups	TN	FP	Specificity (95%CI)	p-value*
**Age groups**				
≤ 30 years	38	1	97.4 (86.5-99.9)	0.543
> 30 years	81	1	98.8 (93.4-100)	
**Sex assigned at birth**				
Male	78	2	97.5 (91.3-99.7)	0.548
Female	41	0	100 (91.4-100)	
**Time since HIV diagnosis**				
≤ 1 year	41	1	97.6 (87.4-99.9)	1.000
> 1 year	77	1	98.7 (93.1-100)	
**Current ART**				
No	43	1	97.7 (88.0-99.9)	1.000
Yes	76	1	98.7 (93.0-100)	
**CD4 cell count cells/mm^3**				
< 200	20	1	95.2 (76.2-99.9)	
200-500	59	1	98.3 (91.1-100)	0.426
> 500	40	0	100 (91.2-100)	
**Viral load copies/mm^3**				
Undetectable	66	1	98.5 (92.0-100)	
< 1,000	10	0	100 (69.2-100)	1.000
1,000-10,000	11	0	100 (71.5-100)	
> 10,000	32	1	97 (84.2-99.9)	
**History of previous syphilis**				
No	104	1	99.0 (94.8-100)	0.137
Yes	7	1	87.5 (47.3-99.7)	
**RPR titer**				
Non-reactive	118	2	98.3 (94.1-99.8)	
< 1:8	1	0	100 (2.5-100)	1.000
≥ 1:8	0	0	-	

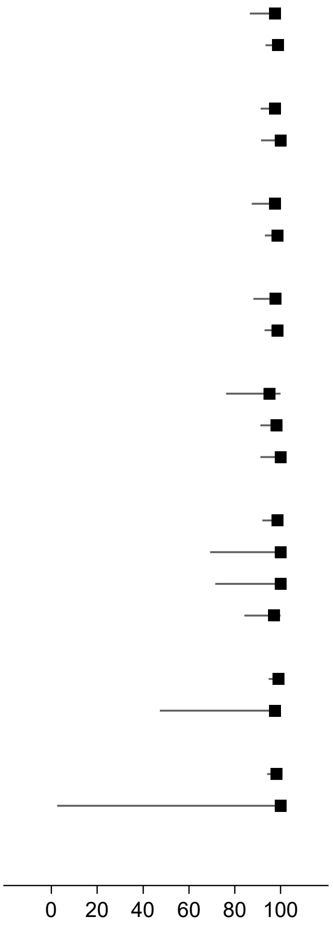

Specificity (%) of SD Bioline in sera compared to TPHA and ELISA in sera from PLWH

*Fisher's exact test

S7 Fig. Specificity of Bioline on sera stratified by demographic, clinical and technical factors.
