# Supplementary material for: Diagnostic performance of two rapid tests for syphilis screening in people living with HIV in Cali, Colombia
Source: PLoS One. 2023 Mar 9;18(3):e0282492. doi: 10.1371/journal.pone.0282492 (PMC9997911; doi:10.1371/journal.pone.0282492)
Supplement: S8 Fig — (PDF) [file pone.0282492.s012.pdf]

Subgroups	TN	FP	Specificity (95%CI)	p-value*
**Age groups**				
≤ 30 years	38	1	97.4 (86.5-99.9)	1.000
> 30 years	79	3	96.3 (89.7-99.2)	
**Sex assigned at birth**				
Male	77	3	96.3 (89.4-99.2)	1.000
Female	40	1	97.6 (87.1-99.9)	
**Time since HIV diagnosis**				
≤ 1 year	42	0	100 (91.6-100)	0.296
> 1 year	74	4	94.9 (87.4-98.6)	
**Current ART**				
No	42	2	95.5 (84.5-99.4)	0.621
Yes	75	2	97.4 (90.9-99.7)	
**CD4 cell count cells/mm^3**				
< 200	19	2	90.5 (69.6-98.8)	
200-500	59	1	98.3 (91.1-100)	0.231
> 500	39	1	97.5 (86.8-99.9)	
**Viral load copies/mm^3**				
Undetectable	65	2	97.0 (89.5-99.6)	
< 1,000	9	1	90.0 (55.5-99.7)	0.492
1,000-10,000	11	0	100 (71.5-100)	
> 10,000	32	1	97 (84.2-99.9)	
**History of previous syphilis**				
No	102	3	97.1 (91.9-99.4)	1.000
Yes	8	0	100 (63.1-100)	
**RPR titer**				
Non-reactive	117	3	97.5 (92.9-99.5)	
< 1:8	0	1	0 (0-97.5)	**0.033**
≥ 1:8	0	0	-	

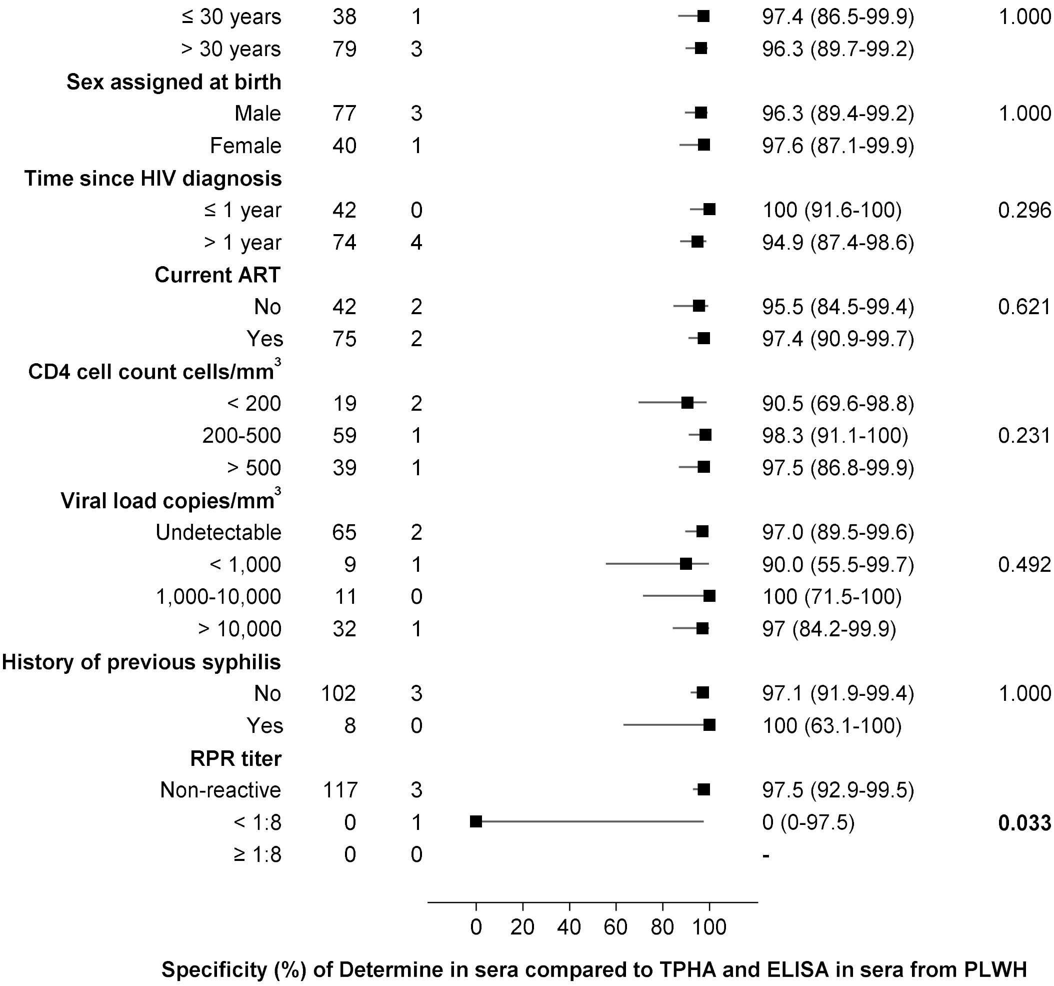

*Fisher's exact test

S8 Fig. Specificity of Determine on sera stratified by demographic, clinical and technical factors.
